# Supplementary material for: Lysosomal Changes in Mitosis
Source: Cells. 2022 Mar 3;11(5):875. doi: 10.3390/cells11050875 (PMC8909281; doi:10.3390/cells11050875)
Supplement: Supplementary file 1 [file cells-11-00875-s001.zip › cells-1606236-SM.pdf]

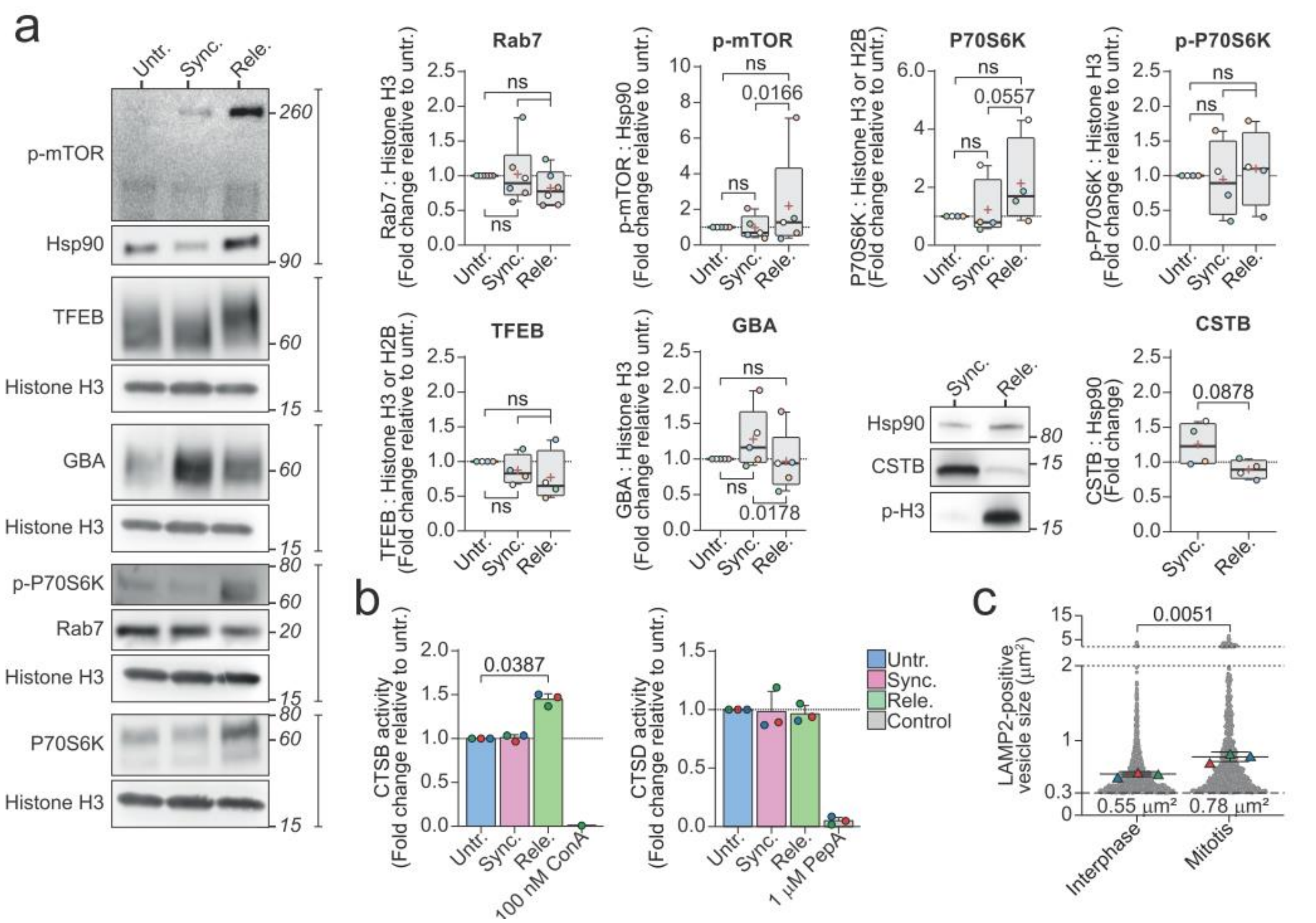

Supplementary figure S1. Western blotting, enzymatic assays, and measurement of LAMP2-postive vesicle size. **(a)** Rep-resentative immunoblotting of lysosomal proteins and CSTB in untreated, synchronized, and released U2OS cells and the quantification of band intensities normalized to respective loading controls, showed as fold change vs untreated. (**b)** Measurement of cathepsin B and D activity in untreated, synchronized, and released U2OS cells. Pepstatin A and ConA were used as positive controls for CTSD and CTSB activity, respectively. (**c**) Measurement of individual LAMP2-positive vesicle area in asynchronous U2OS cells, performed as in figure 2b but using another particle size cutoff-value of 0.3 µm²-infinity. The mean LAMP2-positive vesicle size is shown beneath each plot. All experiments were performed at least three times (n≥3) and different colors symbolize each biological replicate. Statistical analyses were performed on the average values using paired (**a**) or unpaired (**b**) t-test. *P*-values are stated in the figures. Abbreviations: ConA, concanamycin A; CSTB, Cystatin B protease inhibitor; CTSB, cathepsin B; CTSD, cathepsin D; GBA, β-glucocerebro-sidase; LAMP1/2, lysosomal associated membrane protein 1/2; mTOR, mechanistic target of rapamycin; PepA, Pepstatin A; P70S6K, ribosomal protein S6 kinase; p-H3, phospho-S10-histone H3; TFEB, transcription factor EB; V-ATPase, vac-uolar type ATPase.

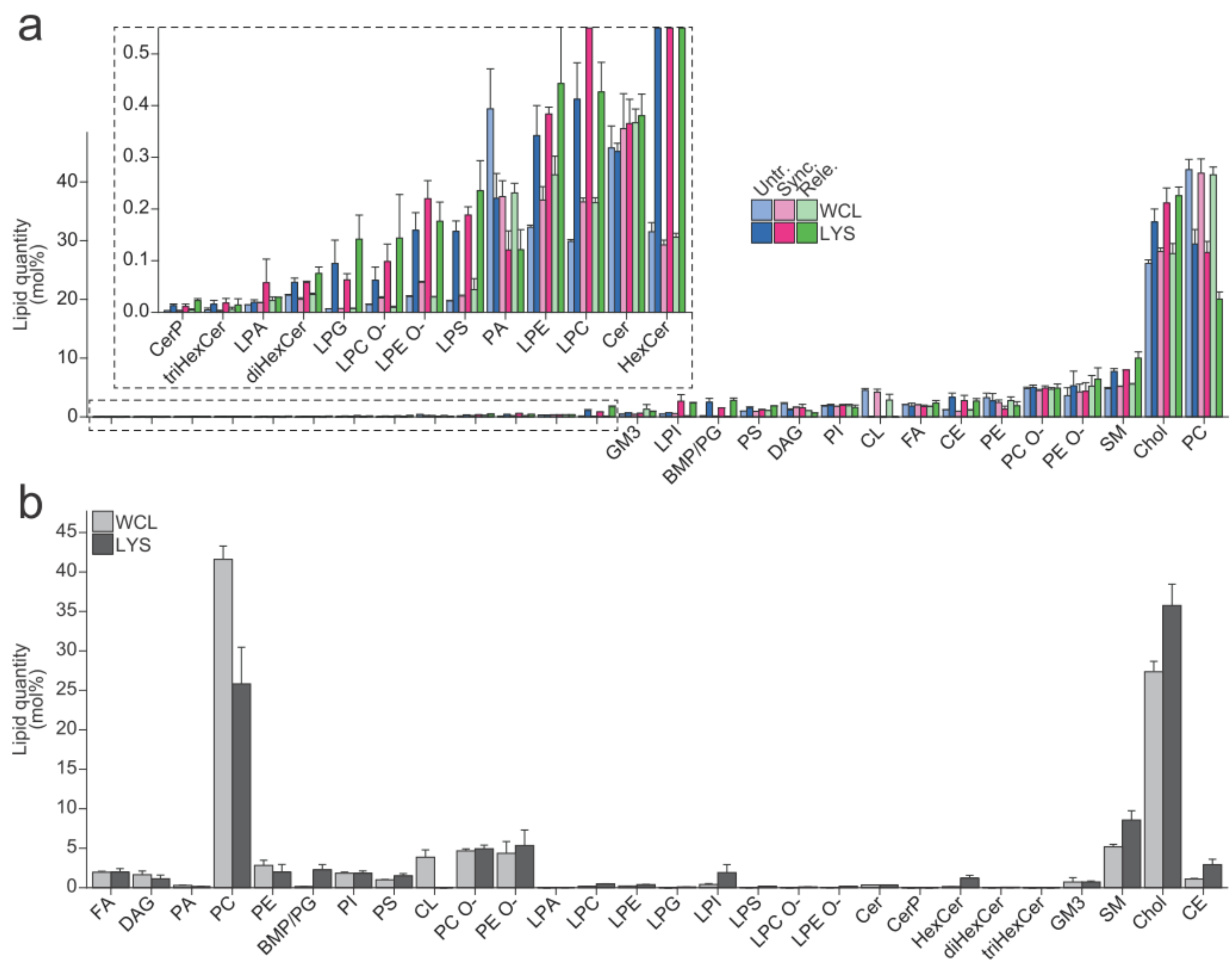

Supplementary figure S2. Quantities of lipid classes. (**a**) Quantities of lipid classes determined for all sample types of whole cell lysates (WCLs) and lysosomal fractions (LYSs). (**b**) Quantities of lipid classes averaged for all WCLs and all LYSs.

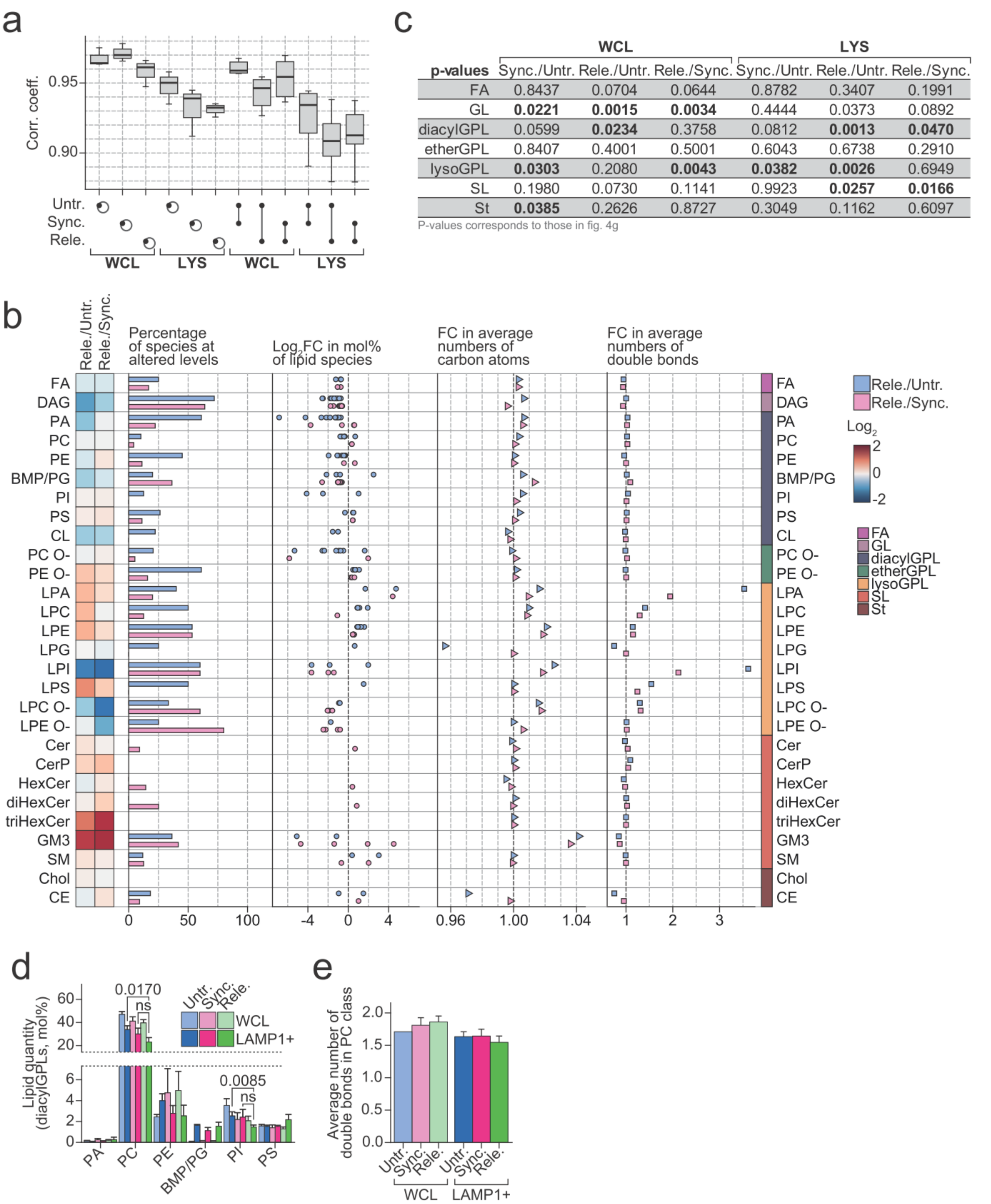

p-values	WCL Sync./Untr.	WCL Rele./Untr.	WCL Rele./Sync.	LYS Sync./Untr.	LYS Rele./Untr.	LYS Rele./Sync.
FA	0.8437	0.0704	0.0644	0.8782	0.3407	0.1991
GL	**0.0221**	**0.0015**	**0.0034**	0.4444	0.0373	0.0892
diacylGPL	0.0599	**0.0234**	0.3758	0.0812	**0.0013**	**0.0470**
etherGPL	0.8407	0.4001	0.5001	0.6043	0.6738	0.2910
lysoGPL	**0.0303**	0.2080	**0.0043**	**0.0382**	**0.0026**	0.6949
SL	0.1980	0.0730	0.1141	0.9923	**0.0257**	**0.0166**
St	**0.0385**	0.2626	0.8727	0.3049	0.1162	0.6097

P-values corresponds to those in fig. 4g

Supplementary figure S3. Additional analyses of lipidomics data. (**a**) Spearman correlation coefficients between replicas or between different sample types calculated based on the acquired quantitative lipid profiles as in figure 4a, but grouped into individual comparisons. (**b**) Lipid profiles of whole cell lysates (WCLs) of released cells relative to controls. *From left to right*: A heatmap of fold changes ($\log_2$) of lipid classes; percentage of lipid species in each lipid class found at statistically significantly different levels by linear modelling; fold change ($\log_2$) estimated for the significantly

changed lipid species; fold change of average number of carbon atoms in each lipid class; fold change of average number of double bonds in each lipid class. (**c**) *P*-values presented and omitted in figure 4g. (**d**) Levels of diacylGPL classes in WCLs and purified LAMP1-positive compartments. (**e**) Average number of double bonds in the lipid class PC in WCLs and purified LAMP1-positive compartments. All experiments were performed three times (n=3). Abbreviations: BMP, bis(monoacyl)glycerophosphate; LAMP1, Lysosomal-associated membrane protein 1; PA, phosphatidic acid; PC, phosphatidylcholine; PE, phosphatidylethanolamine; PG, phosphatidylglycerol; PI, phosphatidylinositol; PS, phosphatidylserine.

Table S1. Antibody information

Antibody	Species	Identifier	Dilution
ASM	Mouse	Abcam #ab74281	1:1000
CTSB	Mouse	Sigma #C6243	1:1000
CTSD	Mouse	Abcam #ab6313	1:1000
EEA1	Rabbit	Abcam # ab2900	1:1000
GBA	Rabbit	Sigma #G4046	1:1000
Golgin97	Rabbit	Abcam # ab84340	1:2000
Histone H3	Rabbit	Abcam # ab18521	1:1000
Hsp90	Mouse	BD transduction #610418	1:1000
LAMP1	Rabbit	Abcam #ab24170	1:500
LAMP2	Mouse	DSHB #H4B4-c	1:1000
mTOR	Rabbit	Cell signaling #2972	1:1000
PDI	Rabbit	Abcam # ab3672	1:1000
P70S6K	Mouse	Cell signaling #9202	1:1000
p-S10-histone H3	Rabbit	Cell signaling #3377	1:5000
p-S2448-mTOR	Rabbit	Cell signaling #2971	1:1000
p-T389-P70S6K	Rabbit	Cell signaling #9206	1:1000
Rab7a	Rabbit	Abcam #ab137029	1:1000
TFEB	Rabbit	Cell signaling #4240S	1:1000
Tom20	Rabbit	Santa cruz # sc-11415	1:3000
VDAC	Rabbit	Cell signaling # 12454S	1:1000
V-ATPase V1 B1/2	Mouse	Santa cruz # sc-271832	1:1000
V-ATPase V0 d1	Rabbit	Abcam #ab202899	1:2000
Rabbit anti-mouse IgG HRP-coupled Secondary Antibody		Dako #P0260	1:10000
Goat anti-rabbit IgG HRP-coupled Secondary Antibody		Thermo Fischer Scientific #31470	1:10000
Rabbit anti-rat IgG HRP-coupled Secondary Antibody		Dako #P0450	1:10000

Table S2. Internal lipid standards

Lipid class	Sum formula	Source	ID	Amount added (pmol)
FA	FA 16:0-D4	TRC-Canada	P145502	24
DAG	DAG 12:0/12:0	Avanti	800812	8
TAG	TAG 17:0/17:0/17:0	Larodan	33-1700	24.39
PA and PA O-	PA 12:0/12:0	Avanti	840635	16.34
PC and PC O-	PC 12:0/12:0	Avanti	850335	20
PE and PE O-	PE 12:0/12:0	Avanti	850702	20
PG and PG O-	PG 12:0/12:0	Avanti	840435	11.03
BMP	BMP 14:0/14:0	Avanti	110857	12
PI and PI O-	PI 8:0/8:0	Avanti	850181	10.89
PS and PS O-	PS 12:0/12:0	Avanti	840038	6.63
CL	CL 14:0/14:0/14:0/14:0	Avanti	710332	20
LPA and LPA O-	LPA 17:0	Avanti	11067	17.64
LPC and LPC O-	LPC 12:0	Avanti	855475	16
LPE and LPE O-	LPE 13:0	Avanti	110696	17.36
LPG and LPG O-	LPG 17:1	Avanti	858127	10.46
LPI and LPI O-	LPI 13:0	Avanti	110716	11.28
LPS and LPS O-	LPS 17:1	Avanti	858141	14.72
Ceramide-1-phosphate (CerP)	CerP 18:1;2/12:0;0	Avanti	860531	16
Ceramide (Cer)	Cer 18:1;2/12:0;0	Avanti	860512	16
SHexCer	SHexCer 18:1;2/12:0;0	Avanti	860573	16
HexCer	HexCer 18:1;2/12:0;0	Avanti	860543	20
diHexCer	diHexCer 18:1;2/17:0;0	Avanti	860595	9.84
triHexCer	triHexCer18:1;2/17:0;0	Larodan	56-1061	12
GM3	GM3 18:1;2/18:0;0-D3	Larodan	71-1107	26
GM2	GM2 18:1;2/18:0;0-D3	Larodan	71-1200	26
GM1	GM1 18:1;2/18:0;0-D3	Larodan	71-1101	26
LHexCer	GluSph-$^{13}C_6$ 18:1;2	Larodan	78-4018	16
LSM	LSM 17:1;2	Avanti	110752	16
SM	SM 18:1;2/12:0;0	Avanti	860583	13.62
Cholesterol	Chol-D4	QMX	D-6359	196.25
CE	CE-D7 15:0	Avanti	700144	17.78

Table S3. Precursor ion, fragment ion and neutral loss for lipid identification

Lipid class	Mode	MS1: Precursor ion	MS2: Fragment ion	MS2: Neutral loss	MS2: *m/z*	MS2: Species specifics
FA	NEG	$[M–H]^-$				
DAG	POS	$[M+NH_4]^+$		[Fatty acid – H + NH_4]		All
PA, PA O-, LPA, LPA O-	NEG	$[M–H]^-$	[Glycerophosphate – H – $H_2O]^-$		152.9958	All
		$[M–H]^-$	[Fatty acid – $H]^-$		*	PA, PA O-, LPA
		$[M–H]^-$	[Fatty acid O- – $H]^-$		*	PA O-
PC, PC O-, SM, LSM	POS	$[M+H]^+$	[Phosphorylcholine + $H]^+$		184.0733	All
LPC, LPC O-	NEG	$[M–H]^-$	[Fatty acid – $H]^-$		*	LPC
PE, PE O-, LPE, LPE O-	NEG	$[M–H]^-$	[Ethanolaminephosphate – H – $H_2O]^-$		196.038	All
		$[M–H]^-$	[Fatty acid – $H]^-$		*	PE, PE O-, LPE
		$[M–H]^-$	[Fatty acid O- – $H]^-$		*	PE O-
BMP/PG, LPG, LPG O-	NEG	$[M–H]^-$	[Glycerophosphate – H – $H_2O]^-$		152.9958	All
		$[M–H]^-$	[Fatty acid – $H]^-$		*	BMP/PG, LPG
PI, PI O-, LPI, LPI O-	NEG	$[M–H]^-$	[Glycerophosphate – H – $H_2O]^-$		152.9958	All
		$[M–H]^-$	[Inositolphosphate – H – $H_2O]^-$		241.0119	All
		$[M–H]^-$	[Fatty acid – $H]^-$		*	PI, PI O-, LPI
		$[M–H]^-$	[Fatty acid O- – $H]^-$		*	PI O-
PS, PS O–, LPS, LPS O–	NEG	$[M–H]^-$	[Glycerophosphate – H – $H_2O]^-$		152.9958	All
		$[M–H]^-$		$[C_3H_5NO_2]$ $\Delta m/z$: 87.032		All
		$[M–H]^-$	[Fatty acid – $H]^-$		*	PS, PS O-, LPS
		$[M–H]^-$	[Fatty acid O- – $H]^-$		*	PS O-
CL	NEG	$[M1–2H]^{2-}$	[Fatty acid – $H]^-$		*	All
Cer, HexCer, di-HexCer, triHexCer	POS	$[M+H]^+$	[LCB + H – $H_2O]^+$		*	All
		$[M+H]^+$	[LCB + H – $2H_2O]^+$		*	All
LHexCer	POS	$[M+H]^+$	[LCB + H – $2H_2O]^+$		*	All
CerP	NEG	$[M–H]^-$	[Phosphoric acid – H – $H_2O]^-$		78.959	All
SHexCer	NEG	$[M–H]^-$	$[HO_4S]^-$		96.9601	All
GM3, GM2, GM1	NEG	$[M–H]^-$	[NeuAc – $H]^-$		290.0864	All
CE	POS	$[M+NH_4]^+$	[Chol – NH_3 – $H_2O]^+$		369.3516	All
Chol	POS (SIM/ tPRM)	$[M+NH_4]^+$	[Chol – NH_3 – $H_2O]^+$		369.3516	All

* Depends on species.
